# The effectiveness of nirsevimab in reducing the burden of disease due to respiratory syncytial virus (RSV) infection over time in the Madrid region (Spain): a prospective population-based cohort study

**DOI:** 10.3389/fpubh.2024.1441786

**Published:** 2024-08-16

**Authors:** José Francisco Barbas Del Buey, Jesús Íñigo Martínez, María Ángeles Gutiérrez Rodríguez, Marcos Alonso García, Amaya Sánchez-Gómez, María Dolores Lasheras Carbajo, Susana Jiménez Bueno, María Dolores Esteban Vasallo, María Alejandra López Zambrano, Cristina Calvo Rey, Manuel Sanchez Luna, Marta Molina Olivas, María Araceli Arce Arnáez

**Affiliations:** ^1^FIIBAP Fundación para la Investigación e Innovación Biosanitaria de Atención Primaria, Madrid, Spain; ^2^Dirección General de Salud Pública, Consejería de Sanidad de la Comunidad de Madrid, Madrid, Spain; ^3^Hospital Infantil La Paz, Madrid, Spain; ^4^University Hospital La Paz Research Institute (IdiPAZ), Madrid, Spain; ^5^Autonomous University of Madrid, Madrid, Spain; ^6^Neonatology Division, University Hospital Gregorio Marañón, Madrid, Spain; ^7^Complutense University of Madrid, Madrid, Spain

**Keywords:** cohort study, respiratory syncytial virus, respiratory syncytial virus vaccines, nirsevimab, infants, effectiveness, intensive care units, hospitalized

## Abstract

**Introduction:**

Respiratory syncytial virus (RSV) infection is one of the main causes of morbidity and mortality from lower respiratory tract infections in children under 5 years of age worldwide. Given that, the objective of this study was estimate the effectiveness of nirsevimab (a single-dose, long-acting, human recombinant monoclonal antibody against RSV) over time for the prevention of respiratory episodes treated at different levels of care.

**Methods:**

A prospective and dynamic population-based cohort study was performed including infants born between April 1 and December 31, 2023, in the Madrid region who resided there during the follow-up period from October 1, 2023, to February 29, 2024. Infants were considered immunized from the day after receiving one dose (50 or 100 mg) of nirsevimab or nonimmunized individuals if they did not receive any dose.

**Results:**

There were 4,100 episodes of primary care, 1,954 hospital emergencies, and 509 admissions, 82 of which required intensive care in the 33,859 participants analyzed. The adjusted effectiveness of nirsevimab in preventing hospitalization due to RSV infection was 93.6% (95% CI: 89.7 to 96.1) at 30 days and 87.6% (95% CI: 67.7 to 95.3) at 150 days. The number needed to treat to prevent one hospitalization were 314.19 (95% CI: 306.22 to 327.99) at 30 days and 24.30 (95% CI: 22.31 to 31.61) at 150 days. The adjusted effectiveness of nirsevimab in avoiding admission to an intensive care unit was 94.4% (95% CI: 87.3 to 97.5) at 30  days and 92.1% (95% CI: 64.0 to 98.3) at 90 days. The adjusted effectiveness of nirsevimab for avoiding primary care consultations and hospital emergency visits was lower.

**Discussion:**

Immunization with nirsevimab is an effective measure for reducing the burden of care related to RSV at all levels of care albeit it decreases throughout follow-up. At 150 days it remained high for preventing hospital admissions. Other articles already published have also demonstrated high effectiveness although with preliminary results, short follow-up periods and wide confidence intervals. None have detected a decrease in effectiveness over time. These results can be quite useful in individual infant prevention and in the design of immunization campaigns.

## 1 Introduction

Respiratory syncytial virus (RSV) is one of the main causes of morbidity and mortality from lower respiratory tract infections in children under 5 years of age worldwide. It is considered the cause of 70% of cases of bronchiolitis in this age group (1).

The burden of RSV disease in Europe, including Spain, is very high, particularly in children under 1 year of age, and even in healthy children (2–4). In the Madrid region (MR), RSV infections cause significant health care overload every winter (5), both in primary care services and in hospitals, including emergency departments, hospitalization floors, and pediatric intensive care units (PICU). Furthermore, the incidence of urgent admissions among children under 1 year of age in the 2022–2023 season was 5,574.5 cases per 100,000 (6). In Spain from 2018 to 2021, there was a bronchiolitis hospitalization rate of 3,650.29 per 100,000 children under 1 year of age (7).

In October 2022, the first long-lasting monoclonal antibody against RSV, nirsevimab (Beyfortus^®^), was developed (8) for the general population, which, in clinical trials, showed high efficacy and safety against RSV infections in childhood with the administration of a single dose (9, 10). Previously, the monoclonal antibody palivizumab was available for the prevention of RSV only in children under 2 years of age at high risk of severe disease, and monthly administration of palivizumab–generally five doses–is required during the RSV season (11).

Spain was one of the first countries in Europe to introduce a nationally funded program for the prevention of RSV in infants. Thus, in July 2023, the National Immunization Advisory Committee recommended the administration of nirsevimab to infants of less than 6 months at the start of or during the 2023–24 RSV season (12). This recommendation was implemented in the MR, one of the regions in Spain with the largest population (6,871,903 inhabitants), and the immunization campaign for infants was implemented on October 1, 2023 (13).

To date, preliminary results on the effectiveness and impact of the use of nirsevimab in the 2023–2024 season are available from countries such as the US and Luxembourg (14, 15), as well as from some regions of Spain (16–19) whose population sizes are low or whose follow-up times were less than or equal to 3 months.

Considering that this measure was recently presented, we still do not have sufficient data on possible factors related to its effectiveness in preventing the different types of care required according to disease severity or the duration of the effect.

The main objective of this study was to estimate the effectiveness of nirsevimab and its variation over time in the prevention of hospitalization and the use of intensive care due to RSV in the region in participants born between April and December 2023 and followed up during the season from October 2023 to February 2024. The secondary objectives were to estimate the effectiveness of the prevention of episodes requiring primary care due to suspected bronchitis/bronchiolitis (syndromic diagnosis) and the prevention of hospital emergency care for episodes related to RSV.

## 2 Methods

### 2.1 Study design

This was a prospective and dynamic population-based cohort study in which two groups or cohorts were differentiated according to their RSV immunization status.

### 2.2 Population, study setting and participants

A free of charge and universal population immunization campaign aimed at those born from April 1, 2023 to March 31, 2024 with nirsevimab was carried out in the MR. The campaign lasted from October 1, 2023, to March 31, 2024. Immunization was performed on children born between April and September at points of hospital immunization, by appointment, at the beginning of the campaign. Those born after October were offered immunization preferentially before hospital discharge, in public and private maternity wards, with a hospital immunization point being enabled throughout the campaign.

In this study, the cohort of those born in the MR from April 1, 2023 to December 31, 2023 and residents in the same were used as the target population. The study and monitoring period ran from October 1, 2023, to February 29, 2024 (date of administrative censorship).

Infants were considered immunized from the day after receiving one dose of nirsevimab or from the first dose if they received more than one dose. Infants were nonimmunized individuals if they did not receive any dose.

For the population under study, records were collected from the personalized registry of endocrine-metabolic diseases which contains nominal information on new-borns (NBs) who are screened for congenital metabolic diseases, in the MR and its progenitors. This registry contains information on all NBs in both public and private maternity wards in the region. It also contains information on the NB and mother, sociodemographic data and perinatal health data at the time of delivery (type of gestation—singleton or multiple—and weeks of gestation). From the MR vaccination registry, which contains all nirsevimab immunizations that are carried out, the history of immunization and the date of the immunization were obtained, thus allowing the two cohorts of the study to be established.

From this framework were excluded the empty units (deaths, changes in residence outside the MR and losses, all of them prior to the start of the follow-up period) and the extraneous units (stillbirths or abortions, nonresident parents in the region, transient population, duplicates and records not corresponding to individuals due to recording errors). The omissions of the framework population are considered negligible since all NBs are routinely screened for congenital metabolic diseases. Due to the impossibility of having their immunization status and gestational history documented, this framework does not include NBs in territories other than the MR who moved their residence to our community during the study period.

For censorship due to death, the mortality registry -namely, medical certificates of death-, which included all deaths and the causes of the death that occurred in the region, as well as of the residents in the MR that occurred in another region, was consulted.

Subjects who received palivizumab or vaccination for the primary prevention of RSV infection in the mother were excluded from the study.

After the start of the population immunization strategy, individualized epidemiological surveillance of subjects with RSV infection was implemented to evaluate its impact. During the follow-up period, events related to RSV infection were recorded at different levels of care (suspicions of RSV infections that require evaluation in primary care or in hospital emergency care, and hospital and PICU admissions due to confirmed infections), comorbidities and possible losses to follow-up (change of residence to another community or country, death from other causes).

In confirmed cases of RSV infection, an epidemiological survey of the date on symptoms onset, signs and symptoms present, and risk factors or comorbidities was carried out by epidemiologists.

### 2.3 Outcomes

RSV infection was defined as the presence of an acute infection of the lower respiratory tract with obstruction or inflammation, which may be accompanied by a catarrhal phase and the presence of cough, wheezing, hyporexia, respiratory distress (nasal flaring, chest wall retractions, apnea), feeding difficulties, pneumonia and/or sepsis. If more than 14 days had elapsed between two episodes, the child was regarding as having a recurring episode.

- Hospital admission: hospital admission due to clinical manifestations compatible with severe acute respiratory infection, confirmed by PCR or the antigen test or RSV isolation test of a respiratory sample.
- Intensive care episodes: Hospital admission, defined above, in which PICU admission has been required at some point
- Episodes in primary care: clinical suspicions of bronchiolitis with the R78 code of the CIAP-2 of the International Committee for the Classification of Wonca (20).
- Hospital emergencies episodes: clinical suspicions of bronchiolitis with the ICD-10 codes B97.4, J12.1, J20.5 or J21.0 in the first two diagnoses.

### 2.4 Variables

The study factor was the state of immunization with nirsevimab. The infant was considered immunized after 1 day of intramuscular administration of a dose of 50 or 100 mg of nirsevimab (respectively if their weight was less than 5 kg or not). In the survival analysis, the infant was considered immunized during the subsequent follow-up time and nonimmunized during the previous one.

The response variable was the risk rate. To obtain this information, the individual times elapsed (continuous variable) from the beginning of follow-up (date of birth for those born after October 1, 2023, or this date for those born earlier or on the same day) were collected, as were data on the event (RSV infection) or censorship (loss to follow-up, death from another cause or administrative censorship). The event was analyzed according to severity in terms of whether it required assistance from primary care, assistance from the hospital emergency department, hospital admission, or admission to the intensive care unit.

The following control and interaction variables were considered: sex (binary variable) and age in months (continuous variable), with variation according to the follow-up time (updated covariate) on the start date of follow-up, at the time of immunization and at the time of the event or censorship; gestational age at the time of delivery (categorical: 23–27, 28–33, 34–36, 37–41, 42 or more weeks); type of gestation (binary variable, singleton or multiple); presence of comorbidities such as binary variables (if prematurity, heart disease, lung disease, immunosuppression, endocrine disorders, neurological disorders, or need for palliative care occurred); and the mean net income per person in 2021 of the census section of residence (categorical variable were evaluated according to the percentile categories, namely, ≤10th, 11–30, 31–50, 51–70, 71–90 and 91–100).

To assess comorbidities, a search of the records of the computerized medical history of primary care provided by the CIAP-2 of the International Committee for the Classification of Wonca was performed (20), and data were grouped under the following headings: premature infants younger than 35 weeks of gestation (A93); pulmonary pathology (R89, T99 and R99); cardiac pathology (K73, K77 and K82); severe immunodepression or immunosuppressive treatment (A90, B72, B73, B79 and B99); congenital disorders of metabolism (T80); neurological diseases (N99); and the need for palliative care (A99.01). This detection of comorbidities was carried out until March 13, 2024.

Finally, for adjustment according to the temporal and spatial trends during the RSV infection season, the following variables were used: the cumulative incidence of suspected RSV infection in the population aged 0 to 5 years in the residence census section (categorical variable were evaluated according to the percentile categories ≤10th, 11–30, 31–50, 51–70, 71–90 and 91–100) and, as a calendar date, the epidemiological week at the start of follow-up, which indicates the fraction of the epidemiological curve of the study period to which the participants were individually exposed.

### 2.5 Statistical methods

The population frame was refined to eliminate empty or extra units, as well as to correct missing, unreliable or incorrect values. Information on immunological status, comorbidities and corresponding information from the epidemiological survey of confirmed cases of RSV was obtained.

No imputation of values was carried out in the presence of missing values, and these values were excluded from the analysis.

In the analyses of the characteristics of both cohorts, the chi-square test, Fisher’s exact test or t- test was used, depending on whether the variable was a categorical or continuous variable. The log-rank test was used to evaluate differences in the survival function in the different categories of variables, and the trend test of the survival function was used to assess whether the differences showed a trend. In all association analyses, a significance level of 0.05 was used.

The effectiveness of immunization with nirsevimab was measured through survival analysis with respect to the different events of the study (any event due to RSV infection: hospital emergencies, hospital and PICU admissions and primary care consultations). For measurements of crude effectiveness (univariable analysis) and adjusted effectiveness (in the presence of confounders and interactions), a proportional hazards model (21) for the counting process was constructed (22). The adjusted effectiveness estimation was calculated using the formula $\left(1-HR\right)\cdot 100$ .

For the adjustments in the multivariable model, initially, a hierarchical model that included the interactions of the first degree of the control variables described above with the study factor (23) and the interactions of the different independent variables with the time of analysis was established. Nonsignificant interactions with *p* > 0.05 in the likelihood ratio test (LR-test) were eliminated from the model. The final model contained all the possible confounders or control variables described above.

The continuous variables were transformed, if necessary, to achieve a log-linear relationship and interactions with the analysis time to comply with the assumption of proportional hazards.

The number needed to treat (NNT) to prevent one outcome was calculated using the hazard ratio and the probability of survival in the untreated group at the time of analysis (24). To estimate the number of events avoided, the formula $\sum_{i=1}^{5}e_{i}\cdot \left(1-HR_{i}\right)/HR_{i}$ was used where $e_{i}$ and $HR_{i}$ are, respectively, the number of events observed in the immunized cohort and the adjusted hazard ratio (at the middle) in month i=1,⋯,5 of follow-up.

As a sensitivity analysis, the calculation of the E-value is carried out for the different effectiveness results in each of the different events. Given the lack of knowledge of possible confounders, this approach has been chosen because it avoids arbitrariness in the election of the different assumptions that required other types of analysis (25, 26). In addition, the effectiveness calculation has been carried out for confirmed events (hospitalizations and PICU admissions) only for cases diagnosed by PCR or isolation (eliminating those diagnosed by antigen test).

In the residual analysis, Schoenfeld residuals, scaled Schoenfeld residuals, Cox–Snell residuals, deviance residuals and martingale residuals were analyzed. For the assessment of possible influencing points, the following values were examined: likelihood displacement and LMAX influence measures.

For the debugging, manipulation and linking of the data, the Pandas, NumPy, DateTime, Regular expressions, Difflib, RapidFuzz 3.6.2 and Joblib libraries were used in Python 3.11.7; QGIS 3.34.4 was used for the spatial data treatment; and Stata/MP 18.0 from StataCorp LLC was used for the statistical analysis.

### 2.6 Patient and public involvement

Prior to the start of the campaign, several information sessions were held for midwives and pediatricians from the primary and hospital care networks. A https://www.comunidad.madrid/servicios/salud/virus-respiratorio-sincitial-vrs was developed with information for both professionals and the general population. To inform and determine the opinions of the parents, posters and informative brochures were designed and distributed in maternity hospitals and primary care centers, as well as through an online acceptability survey. At the end of the campaign -March 31, 2024-, the global immunization coverage was 87%, which was higher than 95% for those born during the transmission season, reflecting excellent acceptance by health professionals and the population.

## 3 Results

### 3.1 Participants

The study population included 37,617 infants, among which after exclusions (336, 20 of whom were deceased), 37,281 infants were included. Of these infants, it was not possible to know the immune status of 214, so the population eligible for immunization with nirsevimab was 37,067.

There were 34,214 infants for whom it was possible to obtain a complete address in the MR and associate the socioeconomic variables with the infants and the cumulative incidence of cases and suspected cases of RSV in children under 5 years of age in their section of residence. There were no differences in immunization among the excluded infants.

There were 33,859 infants in the analysis of the effectiveness of immunization with nirsevimab, due to the availability of all the values for both the study and control variables.

### 3.2 Description of the population

Table 1 shows the characteristics of the population eligible for immunization with nirsevimab (*N* = 37,067). At the end of the follow-up period, 80.08% (95% CI: 79.67 to 80.49) of the participants were immunized.

**Table 1:** Characteristics of participants eligible in the Madrid region for the nirsevimab effectiveness study at the end of fifth month of follow-up.

Eligible participants for the study of nirsevimab effectiveness (*N* = 37,067)						
	Inmunized against RSV (at the end of follow-up)		Non-immunized (at the end of follow-up)		Total number	*p* value
	Number	Frequency (%)	Number	Frequency (%)		
Immune status at the end of follow-up						
Total	29,684	80.08	7,383	19.92	37,067	
Age in months: Median (IQR)						
Start follow up	0.98 (3.38)		2.85 (3.51)		1.44 (3.67)	<0.001^a^
Final follow-up	5.90 (4.39)		7.61 (3.70)		6.26 (4.46)	<0.001^a^
Gestation weeks						
23–27 weeks	66	0.22	18	0.24	84	0.0001^b^
28–33 weeks	477	1.61	74	1.00	551	
34–36 weeks	1,677	5.65	371	5.03	2,048	
37–41 weeks	27,159	91.49	6,784	91.89	33,943	
42 or more weeks	24	0.08	21	0.28	45	
NA	281	0.95	115	1.56	396	
Type of birth						
Single birth	28,754	96.87	7,239	98.05	35,993	<0.001^c^
Multiple birth	930	3.13	144	1.95	1,074	
Sex						
Female	14,290	48.14	3,641	49.32	17,931	<0.001^d^
Male	15,386	51.83	3,731	50.54	19,117	
NA	8	0.03	11	0.15	19	
Comorbidity						
Undetected	27,347	92.13	6,925	93.80	34,272	<0.001^c^
Detected comorbidity	2,337	7.87	458	6.20	2,795	
Comorbidity groups						
Prematurity (< 35 weeks)	1,820	74.53	330	68.04	2,150	<0.001^d^
Lung pathology	204	8.35	55	11.34	259	0.594^d^
Cardiac pathology	280	11.47	70	14.43	350	0.969^d^
Immunodepression	52	2.13	12	2.47	64	0.815^d^
Metabolism disorders	53	2.17	10	2.06	63	0.421^d^
Neurological pathology	31	1.27	8	1.65	39	0.929^d^
Palliative care	2	0.08	0	0.00	2	0.481^d^
Average personal net income of the census section of residence (2021 data). Percentiles						
Up to 10th (lower)	2,219	7.48	1,210	16.39	3,429	<0.001^b^
11th to 30th	4,944	16.66	1,909	25.86	6,853	
31th to 50th	5,375	18.11	1,464	19.83	6,839	
51th to 70th	5,845	19.69	983	13.31	6,828	
71th to 90th	6,116	20.60	730	9.89	6,846	
91th to 100th (higher)	3,082	10.38	336	4.55	3,418	
NA	2,103	7.08	751	10.17	2,854	
Cumulative incidence of suspected RSV in children aged 0 to 4 years by census section of residence. Percentiles						
Up to 10th (lower)	2,778	9.36	603	8.17	3,381	
11th to 30th	5,635	18.98	1,253	16.97	6,888	
31th to 50th	5,537	18.65	1,263	17.11	6,800	0.5273^b^
51th to 70th	5,405	18.21	1,302	17.64	6,707	<0.001^d^
71th to 90th	5,553	18.71	1,447	19.60	7,000	
91th to 100th (higher)	2,674	9.01	764	10.35	3,438	
NA	2,102	7.08	751	10.17	2,853	

a Wilcoxon rank-sum test.

b Cochran-Armitage test for trend across ordered groups.

c Two-sample test of proportions.

d Chi square test, table 2 × 2 or table r x 2. r = 1, 2, ..., n (number of rows).

NA, not available.

The ages of the immunized and nonimmunized infants at the beginning and end of the follow-up period were different (*z* = 42.3 and *z* = 35.6, respectively, *p* < 0.001 in both cases), with a difference of approximately 2 months.

The percentage of males was slightly higher in the immunized group than in the nonimmunized group, but these differences were negligible.

In the immunized population, there was a higher percentage of multiple births ( $\chi_{1}^{2}$ = 29.39, *p* < 0.001), comorbidities ( $\chi_{1}^{2}$ = 23.64, *p* < 0.001) and prematurity ( $\chi_{1}^{2}$ = 29.87, *p* < 0.001).

The immunized population showed a significant tendency to have a higher average family income per member than the nonimmunized population ( $\chi_{4}^{2}$ = 73.85, *p* < 0.001).

The follow-up time of the eligible population was 163,897.78 person-months. The follow-up time of the population analyzed for adjusted effectiveness was 149,920.3 person-months, with a mean follow-up time of 4.43 months, a median follow-up time of 5 months and a follow-up range of 0.03 to 5 months.

During follow-up, there were 1,536 censures, 30 of which were deaths none due to RSV infection. Of those who died during follow-up, 12 were immunized, five were extremely premature (less than 28 weeks of gestation), and congenital anomalies (*n* = 8) and perinatal pathologies (*n* = 15) predominated among the causes of death.

Table 2 describes the events that occurred in the eligible population. Episodes of RSV infection were less severe among the immunized population (trend test: $\chi_{2}^{2}$ = 44.17, *p* < 0.001) than among the nonimmunized population. Additionally, in the presence of recurrences, there were differences, with a higher number of recurrences among the nonimmunized population (likelihood ratio test—LR: $\chi_{1}^{2}$ = 6.51, *p* = 0.011).

**Table 2:** Events in eligible participants for the nirsevimab effectiveness study during the five-month follow-up in the Madrid region.

Events in eligible participants for the study of nirsevimab effectiveness (*N* = 37,067)						
	Inmunized against RSV at events time		Non-immunized at events time		Total number	*p* value
	Number	Frequency (%)	Number	Frequency (%)		
Care required during the episodes						
Primary care	2,588	67.71	1,512	53.56	4,100	<0.001^a^
Emergency care	1,076	28.15	878	31.10	1,954	
Hospitalization	133	3.48	376	13.32	509	
Intensive care	25	0.65	57	2.02	82	
Recurring episodes of RSV						
Primary care	119	66.85	204	77.86	323	<0.001^b^
Emergency care	59	33.15	58	22.14	117	
Hospitalization	0	0.00	0	0.00	0	NA
Intensive care	0	0.00	0	0.00	0	NA
Age in months of cases: Median (IQR)						
Primary care	5.65 (3.01)		5.26 (3.65)		5.36 (3.48)	<0.001^c^
Emergency care	3.97 (3.88)		4.95 (3.33)		4.33 (3.81)	<0.001^c^
Hospitalization	2.28 (2.89)		4.37 (3.55)		3.39 (3.61)	<0.001^c^
Intensive care	1.74 (1.96)		2.86 (3.70)		2.14 (2.73)	0.0220^c^

a Cochran-Armitage test for trend across ordered groups.

b Chi square test, table 2 × 2 or table r x 2. r = 1, 2, ..., n (number of rows).

c Wilcoxon rank-sum test.

NA, not available.

The median age of the patients requiring emergency care, hospital admission or intensive care decreased with increasing severity of the event, and the median age of the immunized group was lower than that of the nonimmunized group, with differences of 2 months for admission to the hospital and 1 month for admission to the intensive care unit.

The nonimmunized population had more events than the whole population: 177.4% more admissions (376 vs. 135.54), 132.9% more admissions to the intensive care unit (57 vs. 24.47), 72.6% more visits to the emergency department (942 vs. 545.81) and 40.8% more consultations with primary care (1,647 vs. 1,169.90). All these differences were significant (all *p* < 0.001, and their $\chi_{1}^{2}$ values were 605.56, 64.99, 412.22 and 283.51, respectively, according to the log-rank test).

Regarding sex, there were no differences in survival function between the two groups for hospital admissions ( $\chi_{2}^{2}$ = 2.55, *p* = 0.11) or for care in the intensive care unit ( $\chi_{2}^{2}$ = 0.67, *p* = 0.41). On the other hand, the male population required more care in the emergency department (1,217 vs. 1,072.82) and in primary care (2,693 vs. 2,306.17) than the whole population, with increases of 13.4 and 16.8%, respectively (both of which were significant; *p* < 0.001 and with $\chi_{2}^{2}$ = 40.0 and 134.06, respectively).

Babies at 36 weeks of gestation or less presented a higher trend of attention at the different levels of care than the whole population: they had 40.8% more hospital admissions (52 vs. 36.9) and an increase of 134.5% compared to what was expected (14 vs. 5.97) regarding the need for intensive care; they had 19.5% more hospital emergencies than expected (179 vs. 149.74), and 20.8% more need of attention in primary care than expected (387 vs. 320.44). In all the analyses, this difference was significant in the test for trend in survivor functions, with $\chi_{1}^{2}$ equal to 7.39, 6.94, 12.41 and 10.65, respectively, and *p* values of 0.007, 0.008, 0.0004 and 0.001, respectively.

Among babies born from a multiple gestation there were no differences in survival due to the need for hospital admission ( $\chi_{1}^{2}$ = 1.33, *p* = 0.25) or admission to intensive care ( $\chi_{1}^{2}$ = 1.15, *p* = 0.28) with respect to the survival function of the population. There were differences in emergency care ( $\chi_{1}^{2}$ = 9.54, *p* = 0.002) and primary care ( $\chi_{1}^{2}$ = 9.33, *p* = 0.002): the use of emergency services was 39.4% greater than expected (83 vs. 59.52), and that of primary care was 26.7% greater (161 vs. 127.1).

There were significant differences in the survival of infants with comorbidities compared to the whole population: 75.2% more infants required hospital admission (67 vs. 38.24), 191.7% more infants required intensive care (18 vs. 6.17), 43.6% more infants required emergency care (225 vs. 156.71), and 44.8% more infants required primary care (488 vs. 336.99). In all cases, the log-rank test yielded *p* < 0.001 and $\chi_{1}^{2}$ values of 23.4, 24.54, 32.18 and 73.28, respectively.

There was a significant increase in the rate of care in infants who belong to census sections with the cumulative incidence of children aged 0 to 4 years above the 50th percentile, except for admission to the intensive care unit ( $\chi_{1}^{2}$ = 3.93, *p* = 0.05). There were 30.3% more hospital admissions than expected (300 vs. 253.23), 18.4% more hospital emergencies (1,200 vs. 1,013.93) and 37.9% more primary care consultations (2,985 vs. 2,164.81). These $\chi_{1}^{2}$ values were 63.81, 105.37 and 911.81, respectively; all of them had *p* < 0.001.

In the percentiles lower than or equal to 50 of the average family income per member, there was a significant increase in the number of events for all levels of care except for admission to intensive care ( $\chi_{1}^{2}$ = 1.18, *p* = 0.28). Thus, for these income levels, there was a 36.6% increase in hospital admissions (334 vs. 251.92), a 27.7% increase in hospital emergencies (1,285 vs. 1,006.26) and 18.5% increase in primary care (2,541 vs. 2,144.31). The results of the test for trend in survivor functions ( $\chi_{1}^{2}$ ) for the latter were 95.16, 209.15 and 177.75, respectively, and their *p* values were less than 0.001.

### 3.3 Outcomes and effectiveness

**Figure 1:**
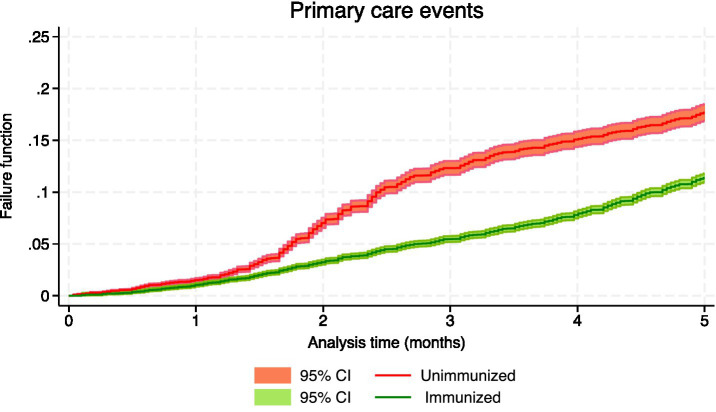
Kaplan–Meier failure estimates that occurred in each cohort in primary care.

**Figure 2:**
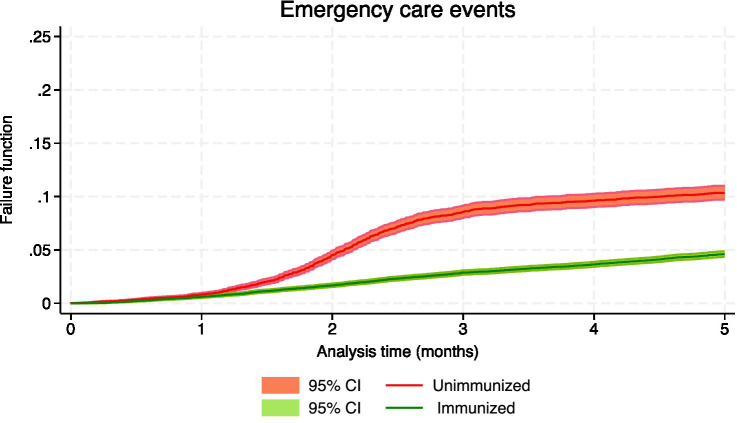
Kaplan–Meier failure estimates that occurred in each cohort in emergency care.

**Figure 3:**
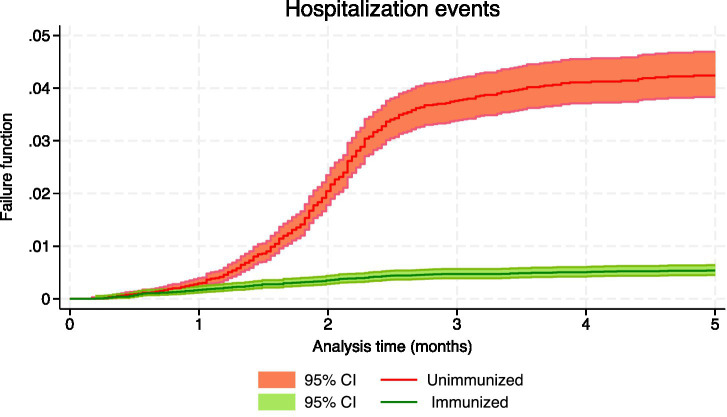
Kaplan–Meier failure estimates that occurred in each cohort in hospitalization.

**Figure 4:**
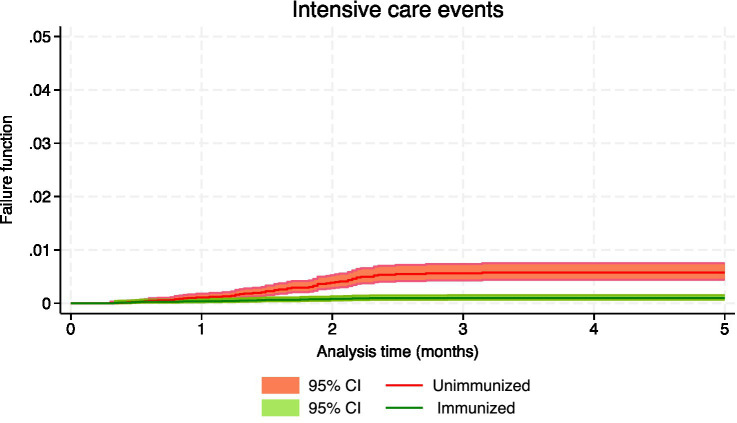
Kaplan–Meier failure estimates that occurred in each cohort in pediatric intensive care units.

Table 3 shows the number of events, the person-months of follow-up and the effectiveness, both crude and adjusted, for the confirmed events of RSV infection, which included hospital admissions and intensive care, as well as suspected events of having RSV, which involved hospital emergencies and primary care consultations. During the 5-month follow-up period, the crude rate of hospitalization was 124.42 cases/100,000 person-months in immunized infants and 863.58 in nonimmunized infants, with an incidence rate ratio (IRR) of 0.14 (95% CI: 0.12 to 0.18); the crude rate of admission to intensive care in immunized infants was 22.49, and in nonimmunized infants was 131.97, being the IRR 0.17 (95% CI: 0.10 to 0.28). Figures 1–4 show the Kaplan–Meier failure estimates that occurred in each cohort in the different types of health care.

**Table 3:** Number of different outcomes, person-months, and crude and adjusted effectiveness of nirsevimab to avoid different care events: confirmed (hospitalization and intensive care events) and suspected (primary and emergency care events).

Immunization effectiveness according to the type of care and follow-up (*N* = 33,859)							
		Immunized		Unimmunized (Reference)		Effectiveness	
	Follow-up	Person-months	Events	Person-months	Events	Crude % (95% CI)^†^	Adjusted % (95% CI)‡
Primary care events ^a^		106,728.09	2,687	43,192.22	1,576		
	First month	16,453.70	199	17,337.43	285	61.7 (57.3, 65.7)	
	Second month	25,441.57	589	8,157.95	490	51.3 (47.5, 54.9)	age modification
	Third month	24,751.25	605	6,772.42	430	38.1 (33.6, 42.3)	(see Table 4)
	Fourth month	21,820.89	559	5,737.09	200	21.3 (13.3, 28.5)	
	Fifth month	18,260.68	735	5,187.33	171	−0.1 (−14.9, 12.9)	
Emergency care events^a^		106,698.63	1,096	43,180.32	908		
	First month	16,455.91	118	17,335.20	139	69.4 (64.4, 73.8)	66.7 (61.0, 71.6)
	Second month	25,442.11	293	8,157.28	325	62.7 (58.7, 66.3)	58.1 (53.5, 62.3)
	Third month	24,749.55	321	6,771.48	327	54.5 (49.4, 59.1)	47.3 (41.2, 52.9)
	Fourth month	21,817.69	177	5,736.45	73	44.5 (34.7, 52.7)	33.8 (21.8, 43.9)
	Fifth month	18,233.37	187	5,179.91	44	32.2 (14.4, 46.4)	16.7 (−5.9, 34.5)
Hospitalization events^b^		106,728.09	132	43,192.22	373		
	First month	16,453.70	34	17,337.43	46	80.4 (73.8, 85.3)	93.6 (89.7, 96.1)
	Second month	25,441.57	50	8,157.95	157	89.0 (86.5, 91.1)	92.5 (89.9, 94.4)
	Third month	24,751.25	33	6,772.42	138	93.8 (91.0, 95.8)	91.1 (86.9, 94.0)
	Fourth month	21,820.89	8	5,737.09	24	96.5 (93.6, 98.1)	89.5 (79.8, 94.6)
	Fifth month	18,260.68	7	5,187.33	8	98.1 (95.4, 99.2)	87.6 (67.7, 95.3)
Intensive care events^b^		106,728.09	24	43,192.22	57		
	First month	16,453.70	7	17,337.43	17	75.7 (57.1, 86.2)	94.4 (87.3, 97.5)
	Second month	25,441.57	12	8,157.95	25	89.6 (79.9, 94.6)	93.3 (85.6, 96.9)
	Third month	24,751.25	5	6,772.42	14	95.5 (83.8, 98.8)	92.1 (64.0, 98.3)
	Fourth month	21,820.89	0	5,737.09	1	98.1 (85.8, 99.7)	90.7 (−3.6, 99.2)
	Fifth month	18,260.68	0	5,187.33	0	99.2 (87.3, 99.9)^c^	89.0 (−207.3, 99.6)^c^

†Simple Cox regression with interaction of immunization with analysis time. ‡Multivariable Cox regression adjusted for sex, age in months, gestational age (categorized), type of delivery, presence of comorbidities (binary), percentile of average income per person (categorized) in their census section of residence, cumulative incidence of suspected RSV in the population under 5 years of age in their census section of residence (categorized) and the calendar week of the start of follow-up.

a Suspected RSV infection.

b Confirmed cases of RSV infection.

c Not interpretable due to lack of events (estimate out of range).

Since immunization is a variable that changes during follow-up, simple Cox regression was used to estimate crude effectiveness. In the evaluation of the different types of events, the interaction of immunization with the analysis time was detected, and this indicated that the effect of immunization on the incidence function varies over time. The $\chi_{1}^{2}$ results of the proportional hazards assumption test for hospital admissions, intensive care admissions, emergency care and primary care consultations were 16.26, 3.95, 26.02 and 92.03, respectively, corresponding to *p* values of 0.001 and 0.047, respectively, for the first two and less than 0.001 for the latter two.

### 3.4 Adjusted effectiveness evaluation

The adjusted effectiveness of nirsevimab was evaluated by multivariable Cox regression, with adjustments for the six control variables indicated in the “Variables” subsection of the Methods section and as variables to control for possible differences in exposure to RSV during the course of follow-up: the calendar week of initiation of follow-up and the cumulative incidence of RSV for children under 5 years of age in their census tract of residence.

For all the events analyzed, except for emergency care (link test or the prediction squared: *z* = −1.25, *p* = 0.21), the age variable did not fulfill the assumption of log-linearity, so it had to be transformed with fractional polynomials to fulfill this relationship. The best transformation showed its advantage over the linear relationship based on the deviance difference for hospital admissions ( $\Delta D_{3}^{2}$ = 13.82, *p* = 0.003), for intensive care admissions ( $\Delta D_{4}^{2}$ = 8.89, *p* = 0.064) and for assistance in primary care ( $\Delta D_{5}^{2}$ = 17.1, *p* = 0.004).

Most of the interactions of immunization status with the different covariates were not significant. The exception was the attention of primary care, for which the interaction was significant with the transformed age (LR-test: $\chi_{4}^{2}$ = 91.95, *p* < 0.001). The calendar week interacts with the analysis time in all the events studied.

The adjusted effectiveness are shown in Table 3. They varied with the follow-up time, leading to a strictly decreasing monotonic sequence.

The effectiveness of nirsevimab in preventing hospital admission for RSV was 93.6% (95% CI: 89.7 to 96.1) at 30 days and 87.6% (95% CI: 67.7 to 95.3) at 150 days. The NNTs to avoid additional hospital admission was 314.19 at 30 days of follow-up (95% CI: 306.22 to 327.99), 45.29 at 60 days (95% CI: 44.36 to 46.59), 26.31 at 90 days (95% CI: 25.49 to 27.61), 24.56 at 120 days (95% CI: 23.22 to 27.61) and 24.30 at 150 days (95% CI: 22.31 to 31.61). The estimated number of hospital admissions prevented during follow-up was 1,716.1 (95% CI: 1,054.4 to 2,797.4).

Similarly, the effectiveness of avoiding the need for intensive care due to confirmed RSV infection was 94.4% (95% CI: 87.3 to 97.5) at 30 days and 90.7% (95% CI: −3.6 to 99.2) at 120 days (the results in the fifth month are not interpretable due to the absence of events).

The effectiveness of avoiding primary care visits for suspected RSV, in addition to the modification of the analysis time, was modified by age, as described in Table 4. The effectiveness of avoiding the need for hospital emergency care was 66.7% (95% CI: 61.0 to 71.6) at 30 days and 16.7% (95% CI: −5.9 to 34.5) at 150 days.

**Table 4:** Adjusted effectiveness against primary care events (suspected RSV infection).

Immunization effectiveness (*N* = 33,859)		
Effectiveness against primary care events		
Age	Follow-up	Adjusted % (95% CI)
15 days old		
	First month	69.0 (63.5, 73.7)
	Second month	60.9 (55.0, 65.9)
	Third month	50.6 (43.6, 56.7)
	Fourth month	37.5 (27.6, 46.1)
	Fifth month	21.1 (5.5, 34.1)
One month old		
	First month	68.2 (62.6, 73.0)
	Second month	59.8 (54.1, 64.9)
	Third month	49.3 (42.4, 55.3)
	Fourth month	35.9 (26.1, 44.4)
	Fifth month	19.0 (3.5, 32.0)
Three months old		
	First month	59.8 (54.3, 64.6)
	Second month	49.2 (44.4, 53.6)
	Third month	35.8 (30.4, 40.8)
	Fourth month	18.9 (10.0, 27.0)
	Fifth month	−2.4 (−18.7, 11.6)
Five months old		
	First month	48.3 (40.9, 54.9)
	Second month	34.7 (27.6, 41.2)
	Third month	17.5 (9.1, 25.2)
	Fourth month	−4.2 (−17.4, 7.5)
	Fifth month	−31.6 (−54.3, −12.3)^a^

a Not interpretable (estimate out of range).

Analysis of the adjusted effectiveness of nirsevimab in avoiding hospital admissions for confirmed RSV infection by dose revealed a similar decreasing pattern. The effectiveness was 86.1% (95% CI: 50.3 to 96.1) at 150 days of follow-up for the population that received a dose of 50 mg and 85.2% (95% CI: 38.8 to 96.4) for the population that received a 100 mg dose.

The presence of comorbidities does not modify the effect of the intervention analyzed since the interactions between both have not been statistically significant either for hospitalization ( $\chi_{1}^{2}$ = 0.64, *p* = 0.42), or for admission to the PICU ( $\chi_{1}^{2}$ = 1.96, *p* = 0.16), neither for hospital emergencies ( $\chi_{1}^{2}$ = 1.70, *p* = 0.19), nor for primary care assistance ( $\chi_{1}^{2}$ = 3.41, *p* = 0.065). Comorbidities (including prematurity) themselves carry a higher risk of different events: the risk of hospitalizations is multiplied by 1.97 (95% CI: 1.40 to 2.76), by 2.89 for admission to the intensive care unit (95% CI: 1.40 to 5.98), by 1.50 for hospital emergencies (95% CI: 1.2 to 1.85) and by 1.57 for primary care assistance (95% CI: 1.38 to 1.79).

In the quantitative sensitivity analysis, the following E-Value results were obtained: for hospital admissions at 30 days it was 30.84 and its lower bound of 95% CI (lb 95% CI) was 18.88 and at 150 days 15.66 and 5.64 respectively; for PICUs it was 34.97 (lb 95% CI: 15.28) at 30 days and 24.85 (lb 95% CI: 5.00) at 90 days; for emergency episodes it was 5.46 (lb 95% CI: 4.57) at 30 days and 2.39 (lb 95% CI: 1.87) at 120 days.

And finally for the primary care episodes, where an interaction of the study factor with age has been detected, the E-value at 30 days was in the range of 5.91 (lb 95% CI: 4.92) for the 15-days-old children and 3.28 (lb 95% CI: 2.77) for 5-month-old children; and the lowest value of E-Value for those confidence intervals that have significant results has been in the range of 1.85 (lb 95% CI: 1.31) at 150 days for children aged 15 days to 1.72 (lb 95% CI: 1.43) at 90 days for 5-month-old children.

The percentage of diagnoses by PCR or isolation was 63.8% (322/505) in hospitalization events and 66.7% (54/81) for PICU admissions, the rest were confirmed by antigen test. Regarding the sensitivity analysis in confirmed events, taking only into account diagnoses by PCR or isolation, no relevant differences have been observed with respect to the analysis in which antigen tests are included: effectiveness in avoiding hospitalization at 30 days was 91.2 (95% CI: 84.3 to 95.1) and 90.5 (95% CI: 68.3 to 97.2) at 150 days; to avoid admission to the PICU has been 93.2 (95% CI: 81.6 to 97.5) at 30 days and 89.4 (95% CI: 40.3 to 98.1) at 90 days.

In the diagnosis of the statistical models, there were no influential elements except for the need of hospital or intensive care among participants in the immunized cohort. Goodness of fit was determined by checking the coincidence of the observed survival curves (Kaplan–Meier) and predicted by Cox regression, and the verification that the Cox–Snell residuals followed a standard censored exponential distribution with a hazard ratio of one through the cumulative hazard of Cox-Snell residuals graph (showing a slope of one). After the age transformations in the models for primary care, hospitalizations and intensive care admissions, the log-linear relationships were met, with the test for the squared linear predictor *z* = −0.62, *z* = −1.05 and *z* = −0.02, respectively, with corresponding *p* values of 0.53, 0.30 and 0.99. In addition, the interactions with the analysis time were modeled for the variables that did not comply with the proportional hazards assumption.

## 4 Discussion

The present population cohort study offers estimates of the effectiveness of nirsevimab in the largest population cohort published to date (*N* = 33,859) and with a longer follow-up of up to 5 months (150 days). Its effectiveness in the prevention of different health care events in the MR, one of the most populated regions of Spain, was evaluated.

The adjusted effectiveness of nirsevimab in preventing different health care events remains high until the end of the 5-month follow-up, although it shows a decreasing pattern over time, which is consistent with that described in clinical trials (9, 10) and the decrease in the concentration of the monoclonal antibody in the human body over time.

The effectiveness in preventing hospitalization for confirmed RSV infection in children under 6 months of age during the immunization campaign in the region at 30 days was 93.6% (95% CI: 89.7 to 96.1), reaching 87.6% (95% CI: 67.7 to 95.3) at 150 days of follow-up. The NNT to avoid one hospitalization was 314.19 at 30 days (95% CI: 306.22 to 327.99) and 24.30 at 150 days of follow-up (95% CI: 22.31 to 31.61). The effectiveness in preventing the admission of infants with RSV-confirmed infections to intensive care units showed the same pattern, with the effectiveness being 94.4% (95% CI: 87.3 to 97.5) at 30 days and 92.1% (95% CI: 64.0 to 98.3) at 90 days (not enough events were available in the last 2 months of follow-up). The effectiveness in avoiding primary care and hospital emergency visits was lower than that for the remaining events, with values lower than 70% at 30 days.

These data confirm the higher effectiveness of preventing the most severe events (hospitalization and admission to the PICU). However, this lower effectiveness in the prevention of primary care consultations and hospital emergencies may be related to the fact that they are nonspecific suspected diagnoses of lower respiratory tract infections and that could include causative agents other than RSV; the same has been described in another cohort study (18). The real-world studies published thus far (14–19) show similar effectiveness in the prevention of hospitalization, although with smaller populations (greater amplitudes in their confidence intervals) or follow-ups of 90 days or less that do not allow for the detection of a decrease in effectiveness throughout the follow-up observed in our study, attributable to the decrease in the level of antibodies over time (10). The NNT to avoid hospital admission obtained in the present study was similar to that described in other studies, taking into account the differences in follow-up (17, 19).

The preventive effect of immunization with nirsevimab is not modified by the presence of comorbidities, although they carry an increased risk of the different events analyzed.

The crude estimates of effectiveness for the prevention of hospitalization or admission to intensive care units showed that effectiveness was lower at the beginning of the follow-up period. This could be explained by the higher risk of hospitalization due to RSV at a younger age (5).

The effectiveness of preventing visits to primary care is higher in younger children, which could be justified by the described behavior of RSV infections in children under 1 year of age, with a peak of maximum incidence between 2 and 3 months of age (27). This could affect a higher percentage of consultations for RSV with respect to other pathogens in this age group.

Our study also describes differences in terms of sociodemographic variables between immunized and nonimmunized infants, highlighting the higher percentage of multiple births, comorbidities and prematurity in the immunized infant population. In the population with the lowest family income per member, the percentage of nonimmunized infants was higher. These variables have been incorporated into our adjusted effectiveness analysis as well as other cohort studies (18, 19). These findings are of interest for planning strategies for future immunization campaigns.

This study has several limitations. Regarding the study population, since it is necessary to obtain information from different sources and not have a common identifier in all of them, it has not been possible to obtain complete information on all subjects for all variables. Regarding the limitations in the methodology used, it should be noted that in episodes of primary care and emergencies, as the vast majority of episodes are compatible but not confirmed, there might be a misclassification bias that classifies episodes not related to the disease as cases of RSV infection, which leads to an underestimation of effectiveness.

Biases due to unknown confounders are not expected based on the results of the sensitivity analysis. The fact that part of the confirmed diagnoses have been made by antigen testing, which could have given rise to misclassification bias, has not altered the results as demonstrated by the sensitivity analysis carried out.

Our study also has several strengths. First, it uses a large population base that has allowed a robust analysis of effectiveness, adjusted for different factors related to immunization. In addition, the study included both children under 6 months of age at the beginning of the RSV circulation season and those born during the season, which allowed us to control for differences in effectiveness based on the age of the child population. Different outcome variables of interest, namely, primary care consultations, emergencies, hospitalizations and PICU admissions, were included, thus covering the entire spectrum of RSV disease care. Finally, the study period included the entire epidemic season of RSV circulation, and the cumulative incidence of suspected infection reported to the surveillance system and the calendar week of follow-up initiation were used as control variables, which allowed us to control for the intensity of virus circulation throughout the study season.

In conclusion, nirsevimab has a significant impact on the prevention of RSV disease in children under 1 year of age, particularly in terms of the most serious events. The decrease in effectiveness as follow-up progresses should be considered in the design of immunization strategies and supports the relevance of a seasonal campaign coinciding with the time of RSV transmission, as has been done in the 2023–2024 season in the MR. The information derived from this study is of interest to individual infant prevention and justify the continuity of the immunization strategy in our environment and to assess its introduction in other countries with significant disease burden related to RSV.

## Data availability statement

The datasets presented in this article are not readily available because this study was carried out under the legal exception allowed for the investigation and monitoring of health problems in public health surveillance, using identifying data and health records without requesting individual informed consent from the participants. Therefore, for legal and ethical reasons, the underlying dataset is not publicly available.

## Ethics statement

When performing an epidemiological study by institutions with administrative competence in Public Health and in situations of exceptional relevance and seriousness for public health, approval by an ethics committee or the request of individual informed consent or assent are not required according to national legislation (Law 14/1986, of April 25, General Health; Law 33/2011, of October 4, General Public Health; Organic Law 3/2018, of December 5, on the Protection of Personal Data and guarantee of digital rights). This epidemiological study was conducted in accordance with the Declaration of Helsinki, which was revised in 2013. The immunization procedure followed standard practice.

## Author contributions

JB: Conceptualization, Data curation, Formal analysis, Investigation, Methodology, Resources, Supervision, Validation, Writing – original draft, Writing – review & editing. JÍ: Formal analysis, Investigation, Methodology, Writing – original draft, Writing – review & editing. MG: Investigation, Project administration, Writing – original draft, Writing – review & editing. MA: Data curation, Investigation, Writing – original draft, Writing – review & editing. AS-G: Investigation, Project administration, Writing – original draft, Writing – review & editing. ML: Project administration, Writing – original draft, Writing – review & editing. SJ: Data curation, Project administration, Writing – original draft, Writing – review & editing. ME: Data curation, Investigation, Validation, Writing – original draft, Writing – review & editing. ML: Data curation, Writing – original draft, Writing – review & editing. CC: Validation, Writing – original draft, Writing – review & editing. MS: Validation, Writing – original draft, Writing – review & editing. MM: Project administration, Writing – original draft, Writing – review & editing. MA: Conceptualization, Formal analysis, Investigation, Project administration, Supervision, Validation, Writing – original draft, Writing – review & editing.
